# Editorial for the Special Issue on “Experiencing the City: The Relation between Urban Design and People’s Well-Being”

**DOI:** 10.3390/ijerph18052485

**Published:** 2021-03-03

**Authors:** Minou Weijs-Perrée, Gamze Dane, Pauline van den Berg

**Affiliations:** Department of the Built Environment, Eindhoven University of Technology, Vertigo Building, De Groene Loper, Building 6. Postbus 513, 5600 MB Eindhoven, The Netherlands; g.z.dane@tue.nl (G.D.); p.e.w.v.d.berg@tue.nl (P.v.d.B.)

Urbanization brings major challenges with regard to livability and the health and quality of life of citizens. It is recognized that physical design characteristics of a city influence how people experience their surroundings and consequently have an impact on people’s well-being [1]. Therefore, it is important that an urban environment meet the needs of a society so that people have positive experiences, which contribute to their well-being [2,3]. Promoting subjective well-being (SWB) among citizens is one of the fundamental goals of urban sustainability [4].

Furthermore, how a city is experienced (e.g., by seeing, hearing, or feeling) could shape urban life. Because it is recognized that citizens are the most important actors in urban planning [5], there is a growing interest in the relationship between public space and the perception of it by citizens, both in (scientific) research and among policymakers and urban planners. The relation between public space and citizen perception of its accessibility, safety and inclusiveness has also been emphasized at Sustainable Development Goals (SDGs) 11.7 [6]. The consideration of people’s environmental experience and perception is highly important for implementing user-friendly spatial planning [7]. However, there is still little empirical research on the momentary perception, experience, and well-being of people in relation to the urban design of cities.

This special issue contains 16 studies that contribute insights into the relation among urban design, experiences or perceptions and people’s well-being. This editorial summarizes the studies in this special issue. First, the studies’ key findings are described, which focus on different urban public spaces (e.g., parks, green space, walking and cycling environments and public space on campuses) among different age groups. Next, the different (novel) methodological approaches are described. Finally, directions for future research are discussed.

## 1. Types of Public Space

Public spaces in a city can be defined as those that are publicly accessible (e.g., streets, walking or cycling paths, parks, public transportation facilities or public shopping areas) [8,9]. As it is recognized that urbanization is threatening urban greenspace, many researchers stress its importance for people’s well-being [10] and for reducing air pollution and mitigating climate change [11].

Several studies in this special issue focus on urban parks [12,13,14], urban greenspace [15] and urban vegetation [16]. With regard to parks, van Vliet et al. [13] used a non-immersive virtual landscape in a stated choice experiment and via an online survey to collect a large sample (*n* = 697). They estimated the data via mixed logit and latent class models and mainly found that many trees and flowerbeds with different flowers in urban parks are preferred. Benches and a playground are also preferred when people visit an urban park. Two groups of park visitors were distinguished: the nature-loving class (mainly older people, people with disabilities and those with higher education), who value many trees and diverse flowerbeds, and the Amenity-appreciating class (mainly females in good health with children or a part-time job), who prefer furniture, side paths, an absence of litter and a playground.

In addition, Perry et al. [12] used a qualitative approach to analyze the experience of older adults with disabilities, and they argued that parks should be inclusive, inviting and accessible for diverse types of users according to culture, ability, and age). Data were collected by means of focus groups and interviews among older adults with disabilities using the general inductive approach. The participants perceived physical, psychological and social benefits from using a park. They described the importance of good-quality walking paths and park-based exercise equipment, inclusive picnic areas as well as attractive natural elements (e.g., flowers, gardens and birds) and accessible features like bathrooms, seating, play areas and information on park amenities.

In another qualitative study in this special issue on urban parks, Sundevall and Jansson [14] aimed to identify similarities and differences in uses and perspectives of different age groups in urban parks and how their management could create multifunctional social and inclusive parks for all ages. Walking interviews of 18 park users of different age groups (children, adolescents and elderly) were transcribed and qualitatively analyzed. The results showed that children and the elderly use parks (playgrounds and gardens) for socializing, while adolescents lacked a place for socializing in the park. Natural and restorative environments were mainly valued by older adults and adolescents. In addition, all users found cleanliness (no litter), safety, variety of functions/elements and liveliness to be important.

Regarding sound in the urban environment, Krzywicka and Byrka [17], used soundscape recordings in an online survey given under varied conditions, such as visiting while mentally fatigued or engaging in recreational activities. This study found that people preferred a more natural soundscape over an urban one. However, the social context (alone or with someone) did not influence how people perceived the soundscape environment.

The two other studies focused on urban greenspace and proposed new methodologies for urban green design. Veen et al. [15] proposed a new approach for developing evidence-based urban greenspace (UGS) design principles to promote public health, that is, physical health, social cohesion and psychological well-being. In this study, case studies, demographic information, health statistics, residents’ opinions, and data about the current use of UGSs were analyzed to choose the target groups and to formulate health benefit goals. They suggested that support for diverse (physical) activity, and a variety of natural elements, facilities and open spaces was needed. In addition, a mix of open (sub) spaces connected by an interesting network of paths and nodes could increase social cohesion.

As recognized by most studies, vegetation is a valued aspect of urban parks and green spaces (e.g., [12,13] ). Ali et al. [16] developed a pedestrian pathway vegetation tool, which incorporated algorithmic models and a multi-agent system that tested for user friendliness among 20 participants. This tool can be used to find the optimal tree position on a path by incorporating the choices of a variety of stakeholders, including citizens, in decisions about the placement of urban vegetation. This could eventually support a participatory approach for urban decision making.

Other studies in this special issue focus on walking and cycling environments in public spaces and link this with experiences and perceptions such as stress and safety. For example, Llinares et al. [18] analyzed the impact of design variables (number of traffic lanes, natural lighting, artificial lighting and vegetation) on pedestrians perception of safety when crossing a street. In order to measure perceptions of safety, 16 virtual reality scenarios (4 day and 12 night) were tested by means of the psychological and neurophysiological responses of 60 participants. They found that during the daytime people felt safer compared to scenarios at nighttime. Regarding urban design, the results suggested that the number of traffic lanes, nearby vegetation, artificial lighting with color temperature of 4500 K can lead to increased perceptions of safety.

Another study by Liu et al. [19] analyzed the location choice of older adults for leisure-time walking, based on data collected among 316 respondents aged 60 or older in Dalian, China. The results showed that older people with mobility disabilities are less likely use a local square or a park for leisure-time walking. This confirms the importance of creating inclusive parks as proposed by Perry et al. [12] In addition, the results showed that older women especially prefer to walk for leisure in their own neighborhood, and older adults between 70 and 74 prefer to walk in a park.

Using a mixed-methods approach to geospatial analysis, Resch et al. [20] showed that people in city centers of Salzburg and Cologne felt less stressed and more relaxed in close proximity to tourist attractions. People felt more stressed in spaces with more crowded pedestrian areas and at the intersection of traffic lanes (bicycles, cars). This finding is somewhat similar to the finding of Llinares et al. [18] indicating that a higher number of traffic lanes leads to a lower perception of safety. With regard to cycling, they found that a wider bicycle lane leads to less stress compared to a narrow bicycle lane. In addition, the study by Ristea et al. [21], based on a field study of 46 people and a mixed-method approach, showed that people felt less safe at gas stations, in alleys and under bridges or underpasses.

Furthermore, Soares et al. [22] focused on public spaces on Dutch university campuses and showed that proximity to functions (e.g., campus buildings, restaurants, cafés) and environmental features (e.g., greenspace and water vegetation) could lead to more creative encounters. Moreover, Fu et al. [23] investigated the relation between urban vibrancy and man-made environment characteristics via multisource urban spatial information data from an Urumqi case area. As a result of a Geographically Weighted Regression analysis, this study found that land-use intensity and diversity have strong positive effects on urban vibrancy. Fathi et al. [24] found that the characteristics of urban morphology that influence physical activity (spatial safety and accessibility), urban facilities and furniture, spatial integration and connectivity, and environmental comfort were the most important. These findings are similar to those on urban greenspaces by Veen et al. [15]. Also, the aesthetics and visual aspects of the urban environment play an important role in promoting physical health [24].

Finally, Peng et al. [25] analyzed the relationships between people’s attitudes and perceptions regarding the urban environment and outdoor comfort. This study applied structural equation modeling on the data collected from 372 respondents and related meteorological measurements. The results showed that physical thermal exposure condition has a strong effect on people’s comfort assessment. In addition, personal characteristics (e.g., age, income, gender) play an important role in comfort assessment.

## 2. Methods

In the field of urban planning, there is increasing interest in studying episodic and immediate affective states, people’s experiences and their relation with the urban spaces [3,26,27,28]. However, knowledge about the most suitable citizen-driven approach for measuring these affective experiences is still limited.

Besides using traditional methods like questionnaires [19,25], many researchers in this special issue used novel approaches to reveal new methodological possibilities. For example, Resch et al. [20] proposed an evidence-based mixed-method approach for emotion tracking: in this case, of pedestrians and cyclists in city centers. They combined data from wearable sensors (physiological responses) with peoples’ (momentary) perceptions of the environment using an eDiary app, a geolocated (post-hoc paper-and-pencil) questionnaire and data from a wearable video camera (GoPro).

In another study in this special issue, Pykett et al. [29] tried to measure emotional responses to the urban environment using a combination of interviews and bio-sensing data as well as data from an Ecological Momentary Assessment (EMA) Diary and from a daily survey about stress and well-being. They found differences between the objectively measured stress (bio-sensing) data and the subjectively measured (EMA diary and interview) data.

Ristea et al. [21] also used a novel approach to capture different ways that people experience the urban environment. They combined bio-sensing data (moments of stress), with spatial video geonarrative (SVG) objective data on crime rates, population and the built-up environment in addition to data from a survey to analyze perceived urban safety.

The study by Soares et al. [22] combined location data by means of online maps and questionnaires to collect data on people’s momentary perceptions, specifically creative encounters, with urban public spaces at university campuses, which is called Volunteered Geographic Information (VGI). In addition, secondary objective data of the built-up environment (e.g., Openstreet Map) was used.

Studies from Resch et al. [20], Pykett et al. [29], Ristea et al. [21] and Soares et al. [22] showed that combining different data sources (sensors and surveys) and linking them to space via geospatial approaches enhanced citizen engagement in field lab settings and offers potentials for evidence-based planning.

The study by van Vliet et al. [13] used non-immersive virtual environments in an online survey to measure urban park preferences. Furthermore, Birenboim et al. [30] used immersive virtual environments (IVEs) to analyze the influence of the urban environment on walking behavior and well-being. In a pilot study, a walking simulator with a locomotive technology was combined with bio-sensing data, eye tracking and gait sensor data. Moreover, Llinares et al. [18] and Krzywicka and Byrka [17] also made use of virtual urbanscapes and soundscapes on the assessment of surroundings. These studies on virtual environments add another dimension to urban design and well-being, especially for capturing how citizens will experience and perceive new (upcoming) urban design interventions. Thus, urban designers and policy makers can find the optimal solution for healthy and livable areas of future.

Besides these quantitative approaches, several studies also used a more qualitative approach [12,14,15]. These studies offered more insight into the reasons behind the experiences and perceptions in urban environments.

## 3. Future Research

Future work in the field should analyze how people experience urban environments both during the day and at night because these outcomes could differ [18]. Another challenge for future research is to successfully integrate different data collection techniques (e.g., sensoring, surveys, geospatial data collection). In addition, there is still a need for novel forms of analysis in studies that aim to combine objective and subjective data [22,29]. In that sense, volunteered geographic data and social media data can be further investigated [22,23]. Furthermore, including interviews in future research could help improve understanding of the definitional boundaries of emotions, perceptions and experiences related to the urban environment [29]. Using sensor technology and immersive virtual environments (IVEs) in future research could complement traditional diaries and surveys with more dynamic insights into people’s emotional responses and experiences [20,30]. IVE technology still has some limitations, which could be improved in future research [30].

Although there are several directions for future research, this special issue increased our understanding of the relationships between urban design and people’s experiences, perceptions and well-being.

## Data Availability

Not applicable.
